# The Effect of Prestressing and Temperature on Tensile Strength of Basalt Fiber-Reinforced Plywood

**DOI:** 10.3390/ma14164701

**Published:** 2021-08-20

**Authors:** Rynno Lohmus, Heikko Kallakas, Eero Tuhkanen, Volodymyr Gulik, Madis Kiisk, Kristjan Saal, Targo Kalamees

**Affiliations:** 1 Institute of Physics, University of Tartu, W Ostwaldi 1, 50090 Tartu, Estonia; volodymyr.gulik@ut.ee (V.G.); madis.kiisk@ut.ee (M.K.); kristjan.saal@ut.ee (K.S.); 2 Tallinn University of Technology, Ehitajate Tee 5, 12616 Tallinn, Estonia; heikko.kallakas@taltech.ee (H.K.); eero.tuhkanen@taltech.ee (E.T.); targo.kalamees@taltech.ee (T.K.)

**Keywords:** basalt fiber, plywood, reinforcement, layered structures, properties

## Abstract

The reinforcement of plywood is demonstrated by laminating pretensioned basalt fibers between veneer sheets, to fabricate so-called prestressed plywood. Belt type basalt fibers bearing a specific adhesion promoting silane sizing were aligned between veneer sheets with 20 mm spacing and were pretensioned at 150 N. Three-layer plywood samples were prepared and tested for tensile strength at room temperature and at 150 °C. The room temperature tensile tests revealed a 35% increase in tensile strength for prestressed plywood compared to that of the conventional specimen. The reinforcement effect deteriorated at 150 °C but was restored upon cooling to room temperature. The deterioration is attributed to the weakening of bonding between the basalt fibers and phenolic resin matrix at elevated temperatures due to the softening of the resin.

## 1. Introduction

Plywood is one of the main structural materials used in a variety of interior and exterior applications [1]. Nowadays, plywood is one of the most recognized material candidates for structural uses among wood-based panels, because it possesses the advantages of dimensional stability, an excellent strength-to-weight ratio, and high chemical and impact resistance [2]. Therefore, it has traditionally been widely used in light-frame construction and regarded as a trusted building material. In general, the moisture, temperature, wood species, density, grain orientation, veneer quality, number of veneers, and bonding resin influence the overall properties of plywood [3,4,5,6].

Plywood is composed of an odd number of thinly layered (1–3 mm) wooden plies perpendicular to the grain orientation of the previous layer, which makes it a very strong and durable structure [7]. With such features, the mechanical properties of plywood, such as the force–displacement, stress–strain relationship, and failure mechanism are dependent upon the surface-grain orientation [8,9]. Therefore, the grain orientation must be considered when investigating wood or wood-based composite materials.

Odd-layered veneer sheets are glued together by using a proper adhesive. Urea-formaldehyde glue is typically used for indoor applications as it is transparent and the adhesive layer is not visible in cross-sections. In higher humidity conditions, a waterproof adhesive is required. Brown-colored phenol-formaldehyde adhesive forms a strong bond and is thus reliable in outside conditions [10]. In several applications, plywood mechanical and thermal durability is crucial. For example, plywood is a primary construction material in liquefied petroleum gas (LPG) cargo containment systems, where it is constantly exposed to a cryogenic environment and low-frequency vibration. At the same time, plywood weight should be kept as low as possible in order to make room for shipment capacity and reduce fuel consumption. The very same arguments of combining low weight and mechanical durability apply for another mass-use of plywood—trailer vans—which often are made entirely of plywood. Such applications would be the main beneficiaries of using reinforced plywood.

Plywood and other wood-based panels have been successfully reinforced with fibrous additives. Commonly used reinforcement fibers are made of carbon, aramid, or glass [11]. These are linked with either high production costs [12] or problematic recycling [13]. For example, carbon fiber manufacturing has a high energy cost [14]. The production of glass fibers requires additives [15]. This is a tangible difference from basalt fibers. Basalt is directly processed into fibers by melting without the need for modification [16]. The melting temperature of basalt fibers is only slightly higher than that of glass fibers, but their mechanical properties are superior [17]. The life-cycle assessment of overall basalt fiber production confirms a considerably lower environmental footprint than those of carbon or glass fibers [18]. Recycling of basalt fibers is notably simpler because basalt as a material is not modified during the production of fibers. Additionally, sources of basalt are abundant [19]. Nowadays, basalt fibers are of interest for the reinforcement of wood–plastic composites [20,21] and the reinforcement of timber beams [22,23]. They have also been widely used for concrete reinforcement applications [24]

The fiber-reinforced plywood composition has several combination possibilities. One of these cases is carbon fiber and a phenol-resorcinol formaldehyde matrix. However, specimens tested by bending failed due to delamination [25]. The authors explain this behavior with the distinctive mechanical properties of the materials used. Delamination also occurred when strengthening plywood with basalt fiber-reinforced epoxy resin [26]. Several other studies describe approaches to plywood reinforcement. Carbon fibers bonded by ocyanate-based adhesive were used by Ashori et al. [27]. A similar study with chopped carbon fibers and phenol-resorcinol formaldehyde was performed by Xu et al. [28]. Bal et al. [29] reinforced plywood with glass fibers and phenol-formaldehyde adhesive. Auriga et.al. studied two different carbon fiber orientations and two different locations to improve the properties of a standard wood-laminated composite and showed an increase of the static bending strength and modulus of elasticity and a decrease of the tensile strength perpendicular to the planes compared to industrial type 5-ply boards [30]. Biblis et al. investigated plywood strengthening with glass-fiber-reinforced polyester resin [31]. This was applied both between veneers and on the surfaces of plywood. Most of these studies describe a significant improvement in terms of stiffness, flexural strength, and ultimate failure load. The best effect was typically achieved for higher amounts of fibers located closer to the surface of the panel.

Of all the plywood reinforcement studies available today, very little has been reported on reinforcement with basalt fibers. Compared to commonly used glass and carbon fibers, basalt fibers have advantages in industrial-scale use, having better physicomechanical properties than glass fibers and being significantly cheaper than carbon fibers. In this study, the basalt fibers prestressing effect in plywood reinforcement is investigated. The main goal was to improve the mechanical properties of plywood subjected to a tensile load. Prestressing is a well-known method for the reinforcement of building materials, e.g., prestressed concrete, but it has not been reported for plywood. In this work, for the first time pretensioned basalt fiber-reinforced plywood composites’ (i.e., prestressed plywood) tensile strength is studied at different temperatures.

## 2. Materials and Methods

### 2.1. Basalt Fibers

Basalt fibers were obtained from a company called “Kamenny Vek”, Dubna, Russia. The type of chosen fiber was BCF 19/1200 direct roving with KV-42 sizing, a type of silane sizing designed for compatibility with epoxy and phenolic resins [32]. Number 19 in the product code refers to the fiber single strand diameter in micrometers, and 1200 is the linear density (tex) value. According to the manufacturer, the fiber monofilament tenacity is at least 600 mN/tex, which corresponds to 720 N for a macro fiber.

### 2.2. Preparation of Plywood Samples

**Figure 1:**
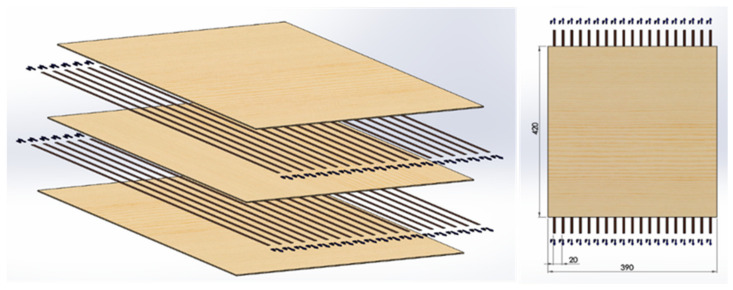
Exploded (**left**) and top view (**right**) of the 3-layer plywood test panels used in this study.

Veneers were kept in the dry storage room at a temperature at 20 °C and relative humidity of 20% in order to keep the veneers’ moisture content in the range of 4.5 ± 1.5%. The density of the Birch veneers was measured as 0.523 g/cm^3^. The veneer and plywood process parameters were 25 °C and a relative humidity of 53%. Testing was carried out in the laboratory environment at 23 °C and a relative humidity of 25%. Basalt fibers were placed in a parallel pattern between the veneer sheets (industry grade birch veneer) with a 20 mm spacing between the fibers (Figure 1). The tensile direction of basalt fibers went along the grain of the middle veneer.

**Figure 2:**
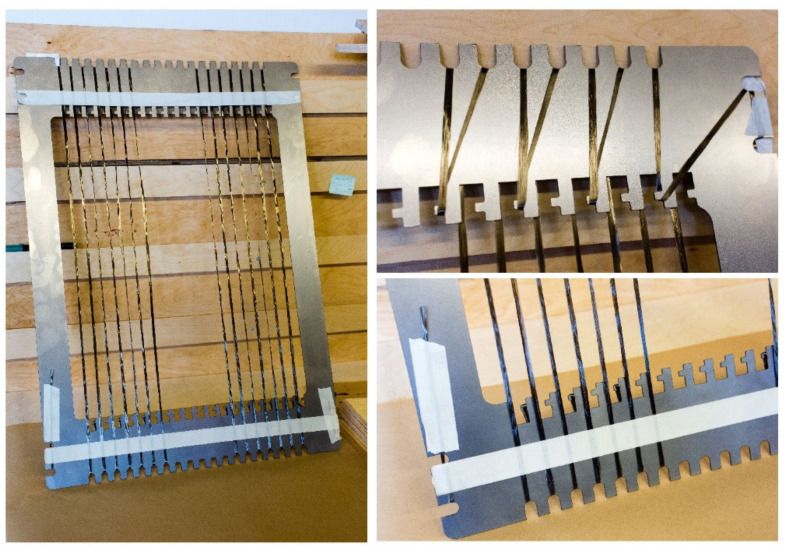
Frame for untensioned fibers alignment. Aligned fibers ready for positioning between the veneer layers (**left**); close-up of the winding layout on the backplane (**upper right**) and of aligned fibers on the front plane (**down right**).

**Figure 3:**
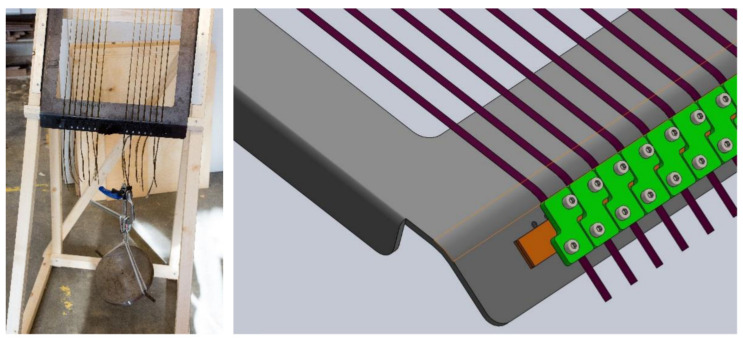
Fiber-tensioning frame. Left image: tensioning with a static load of 150 N per fiber. Right image: schematics of fixing the tensioned fiber. Tensioned fibers (brown) are fixed between two rubber ribbons (orange) by screwing a steel plate (green) tightly against the frame.

All samples were prepared by following the morphological symmetricity requirement for plywood [33]. After composing the lay-up, the 3-ply plywood panels were prepared with a laboratory press, first performing cold pressing at room temperature at 1 MPa for 10 min, followed by hot pressing at 1.8 MPa at 130 °C for 5 min. The veneers were bonded with a commercially available phenolic (PF) adhesive consisting of liquid phenol-formaldehyde resin [34] with a solids content of 49%. Three different sets of plywood samples were prepared: (1) blank plywood (i.e., without fibers), (2) plywood with untensioned (aligned) fibers, and (3) plywood with tensioned fibers. To align the fibers, two sets of specific frames were designed, one for untensioned fibers (Figure 2) and another one for tensioned fibers (Figure 3).

### 2.3. Samples Preparation for Tensile Testing

**Figure 4:**
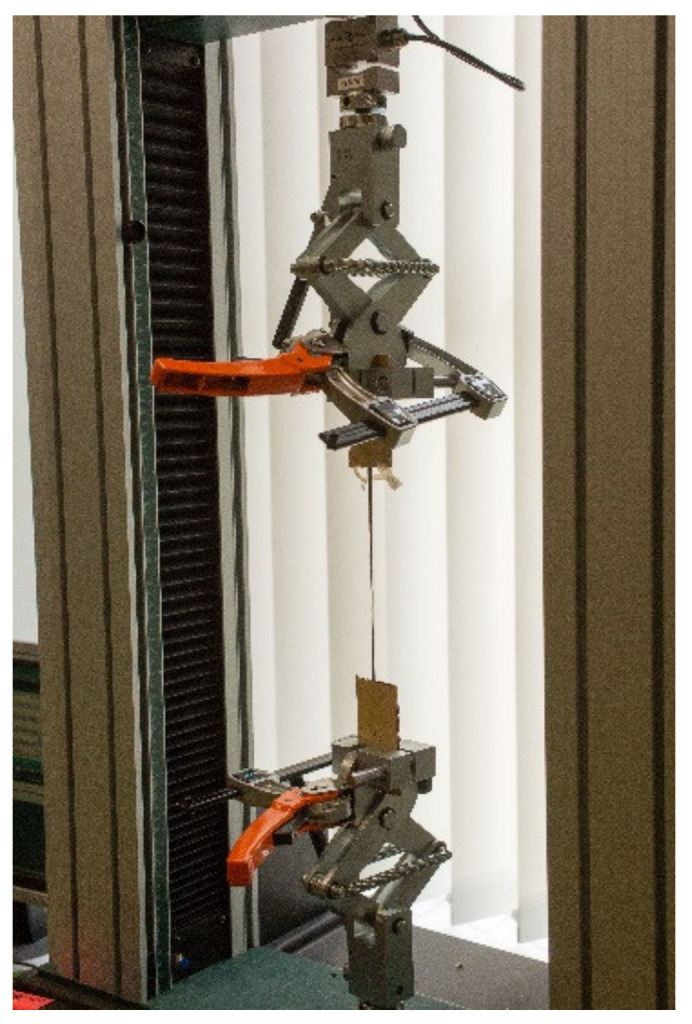
Set-up for the basalt fiber anchorage length and tensile strength measurements.

Two different sets of samples were prepared for tensile measurements. The aim of the first set was to estimate the adhesion and anchorage length of the laminated fibers as well as to measure the tensile strength of a single fiber. For this, both ends of a 200 mm long fiber were glued between 50 mm × 50 mm veneer sheets (Figure 4). The length of the fiber was chosen to match that of the working length of corresponding plywood test samples, which were also subjected to tensile measurements. The anchorage length was varied from 5 to 20 mm. For each anchorage length, six samples were prepared.

**Figure 5:**
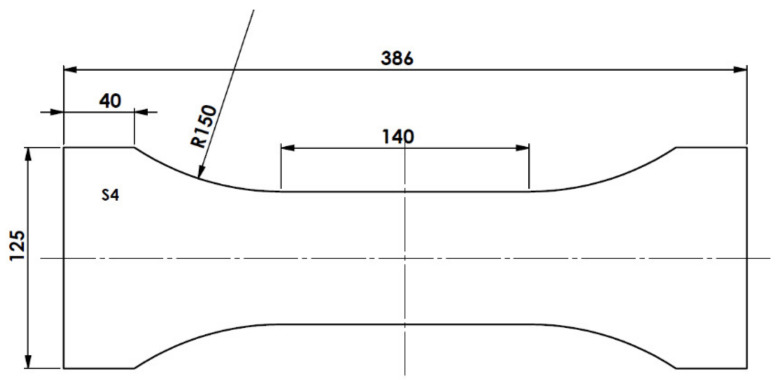
Layout of the plywood tensile testing sample.

Another set of samples were prepared for measurements of the tensile strength of plywood. The samples were shaped according to the wood-based layered materials characterization standard EN 789:2004, however, due to the prepared panel size limitations, the specimen size was proportionally reduced. The layout of the plywood tensile testing samples is presented in Figure 5. Samples with fibers were prepared so that the fibers were parallel to the sample’s bone shape central axis, with each sample containing eight fibers (in two layers).

### 2.4. Tensile Test Measurements

Tensile tests were performed on an Instron 8802 testing system [35]. The tensile rate was 0.0125 mm/min. For precise elongation measurements, an external 100 mm distance extensometer was clamped to the sample.

The tests were performed at two different temperatures. The first set of samples (14 in total) were measured at room temperature (21–23 °C). The second set of samples were heated at 150 °C for six hours and subjected to tensile measurements at this temperature (the specimens were wrapped into aluminum foil-covered rock wool insulation to keep the temperature during the measurements). An additional set of samples were tested for tensile strength after slowly (24 h) cooling back to room temperature.

## 3. Results and Discussion

**Figure 6:**
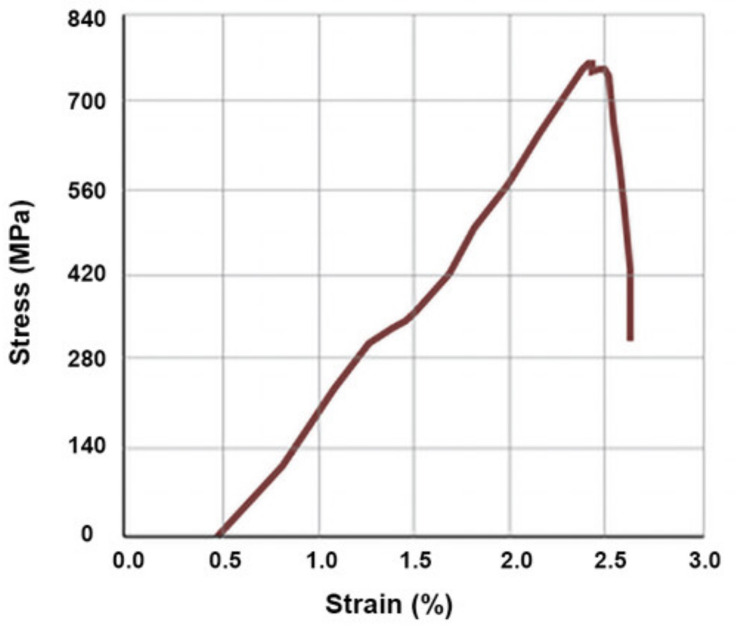
Typical stress-strain curve for basalt fiber at room temperature.

**Figure 7:**
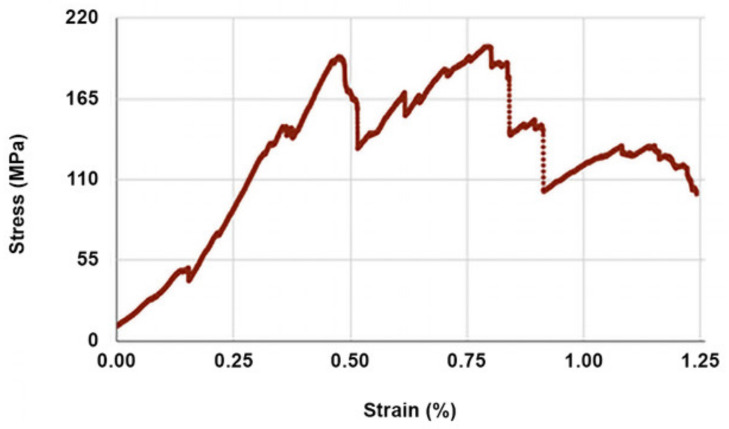
Typical stress-strain behavior of veneer-sandwiched basalt fiber at 150 °C (anchorage length 10 mm).

The anchorage length testing of fibers by tensile measurements revealed that at room temperature an insertion of fiber of even just 5 mm between the veneer sandwich was sufficient to break the fiber without pulling it out of the sandwich. The corresponding average (six samples) load needed to break the fiber was 289 ± 68 N. This is notably less than the calculated 720 N according to the fiber manufacturer’s specifications for a single strand (600 mN/tex), but in such a case an overestimation of the strength of the macro fiber is expected. Figure 6 depicts a typical stress-strain curve of a basalt fiber used in this study at room temperature. The obtained curve is similar in shape to that reported by other authors [33] and shows a ~3% elongation at break. At 150 °C the firm anchorage of fibers was lost, as particularly in the cases of shorter anchorage lengths the fibers were pulled out of the veneer sandwich and the jagged stress-strain curves indicated a slip and stick behavior (Figure 7). Upon slowly (24 h) cooling back to room temperature, the initial tensile characteristics were restored.

**Figure 8:**
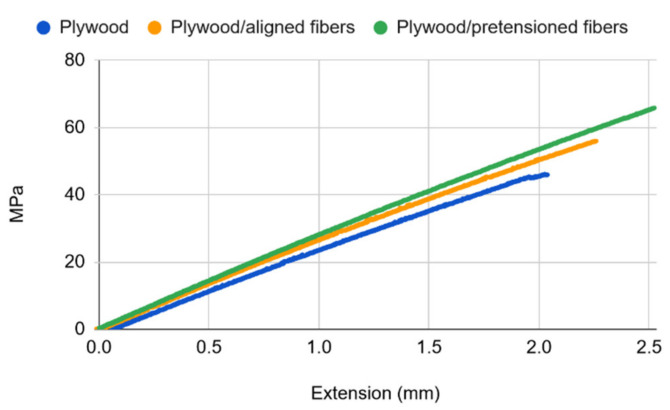
Typical stress-strain relations for plywood and basalt fiber-reinforced plywood at room temperature.

**Figure 9:**
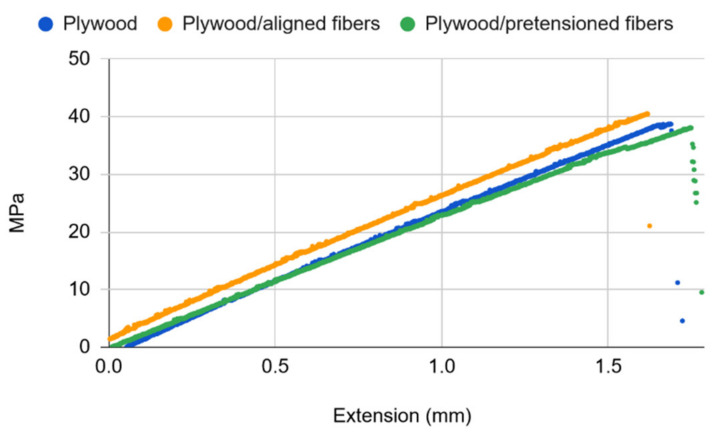
Typical stress-strain relations for plywood and basalt fiber-reinforced plywood at 150 °C.

Figure 8 presents the typical stress-strain relationships for plywood and basalt fiber-reinforced plywood at room temperature. From the plot it can be seen that plywood has a ~1% elongation at break, which is in good agreement with the average number for wood material [36] and which suggests that combining it with basalt fibers that have a ~3% elongation at break would readily allow for the generation of the prestressing effect on plywood at a 50% extension of the fibers. The curves show only a slight difference in stiffness (i.e., slopes), yet clearly indicating that the fiber-reinforced plywood is stiffer, as expected, while at the same time being notably stronger, with untensioned fiber-reinforced plywood yielding a 20% (58.5 ± 3.8 MPa) and pretensioned plywood a 35% (66.1 ± 5.1 MPa) greater ultimate tensile strength than the untreated counterpart (48.7 ± 3.1 MPa). While a similar stiffness to that of untreated plywood seems quite obvious for the untensioned fiber-reinforced plywood, the pretensioned fiber-reinforced plywood showing only a slight increase in stiffness might look unexpected. However, the combined tension of 1200 N exerted by eight fibers in the reinforced plywood specimen only makes about 10% of the ultimate tensile stress of the untreated plywood, meaning that the compressive effect of the pretensioned fibers on plywood and thus on its stiffness is negligible. On the other hand, the increase in the tensile strength of fiber-reinforced plywood cannot be accounted for by the effect of fibers only because plywood is much stronger than the fibers reinforcing it combined (~10 kN for the plywood sample at break vs. ~2.3 kN for the eight fibers combined at break). Consequently, the obtained higher tensile stresses of fiber-reinforced plywood are explained by the suppressed crack evolution in these samples. As would be expected from the results of the anchorage length testing, at 150 °C the reinforcing effect of basalt fibers was completely lost (Figure 9). It is also evident from the plot that the stiffness of the samples was almost identical, as opposed to the slight differences observed for the respective cases at room temperature. The reinforcing effect of basalt fibers on plywood was fully recovered upon slowly cooling to room temperature.

The room temperature mechanical properties’ improvement correlates well with the results by other authors, who have reported basalt fiber reinforcement in the range of 25–40% [37]. Furthermore, the effect of the temperature on the composites has been discussed, but the morphologies of the samples in those works have been very different from those of ours, and thus the relevant conclusions cannot be transposed. It is nevertheless clear that changes in basalt fibers due to temperature can be neglected. The tensile strength of basalt fibers decreases with temperature, but at 150 °C the degrading effect is negligible [38]. The ingredients of wood—hemicellulose, cellulose and lignin—also start to decompose at a higher temperature than that [39], and even if there is some degradation occurring at lower temperatures, this degradation is irreversible and thus cannot account for the results that we obtained. Zhou et al., who studied fiber-reinforced polymers, concluded that the bond weakening of fiber-reinforced polymer bonded wood composites at an elevated temperature was due to the resin matrix softening, which correlated with the glass transition temperature of the resin (Tg) [40]. While specifications for the resin used in the current work do not provide a glass transition temperature, the commercial phenolic resins generally have a Tg of around ~130 °C [41]. Thus, the temperature-dependent reversible tensile strength of basalt fiber-reinforced plywood observed in this work is most plausibly attributed to bond weakening between the basalt fibers and phenolic resin matrix at elevated temperatures.

## 4. Conclusions

This study presents the fabrication and tensile properties of prestressed plywood, which in the case of plywood constitutes a first-time demonstration of simple and cost-effective reinforcement. The reinforcement is achieved by laminating pretensioned (150 N) basalt fibers between veneer sheets with a spacing of 20 mm between the adjacent fibers. At room temperature, the prestressed plywood showed a 35 ± 4.3% increase in tensile strength, as compared to that of the unmodified counterpart (the average of six samples). The adhesion between the fibers and phenolic resin that was used as glue to laminate the veneer was found to be very strong, with an anchorage length of only 5 mm being sufficient to break a fiber during the tensile testing of individual fibers. As the net tensile stress of fibers in the reinforced plywood specimen only makes ~10% of the ultimate tensile stress of the plywood, it is concluded that the basalt fiber reinforcement acts via the suppressing of crack evolution in wood by resisting and damping the applied stress. Upon incubation for 6 h at 150 °C, the reinforcing effect of basalt fibers on plywood was completely lost but was restored upon slow (24 h) cooling back to room temperature. Because of reversibility, the high temperature-related disappearance of fiber reinforcement is likely caused by the softening of phenolic resin at elevated temperatures, thus allowing for the slipping of the fibers with respect to the veneer under stress and thereby preventing them from countering the strain-induced cracking of wood. The obtained results suggest a technologically simple and cost-effective solution for the manufacture of reinforced plywood with only a mere fraction of surplus natural and completely environmentally friendly material in the form of basalt fibers. The temperature tolerance of the reinforcement could be further improved by using high glass transition temperature resins for lamination instead of the conventional phenolic resins used in plywood manufacture.

## Data Availability

Not applicable.
